# Cariprazine in the Treatment of Bipolar Disorder: Within and Beyond Clinical Trials

**DOI:** 10.3389/fpsyt.2021.769897

**Published:** 2021-12-14

**Authors:** André Do, Kamyar Keramatian, Ayal Schaffer, Lakshmi Yatham

**Affiliations:** ^1^Department of Psychiatry, University of British Columbia, Vancouver, BC, Canada; ^2^Department of Psychiatry, Sunnybrook Health Sciences Centre, Toronto, ON, Canada

**Keywords:** cariprazine, treatment, bipolar disorder, clinical trial, *post hoc* analyses

## Abstract

Bipolar disorder (BD) is chronic psychiatric disorder associated with significant impairment in psychosocial functioning and quality of life. Although current pharmacological treatments for BD have improved its clinical management, many patients do not achieve remission, particularly those suffering from bipolar depression. In addition, available treatments are associated with a myriad of potential adverse effects, which highlights the need for novel therapeutic agents that can be effective for both phases of the illness with a reduced side effect burden. Cariprazine is a novel antipsychotic that is a dopamine D2/D3 partial agonist with a preference for D3 receptors. In this review, we examine the pharmacological properties, clinical efficacy and tolerability profile of cariprazine in patients with BD, taking into account the latest clinical trials data. We also review *post hoc* analyses addressing clinically relevant subgroups and symptom domains in BD. Current evidence suggests efficacy for cariprazine 3–12 mg/day in the treatment of acute manic and mixed episodes; for bipolar depression, the efficacy of cariprazine appears to be dose-related, with doses of 1.5–3 mg/day beneficial as monotherapy. Cariprazine is overall well-tolerated by patients in both manic and depressive episodes. Its most common side effects relative to placebo include akathisia, extrapyramidal symptoms and nausea. There are no metabolic concerns reported with cariprazine use. In summary, the latest evidence suggests that cariprazine is an effective and safe treatment option for BD.

## Introduction

Bipolar disorder (BD) is chronic and recurrent psychiatric disorder with a lifetime prevalence of 2.1% (1). It is associated with significant impairment in psychosocial functioning and quality of life, even during periods of euthymia (2). Patients with BD spend up to 50% of their time being ill, with depressive predominating over manic or mixed symptoms (3). The mainstay treatment of BD consists of pharmacotherapy, and specific options depend on the phase of illness (4). Generally, both manic and depressive episodes are managed with mood stabilizers and atypical antipsychotics, either as monotherapy or combination therapy. Although current pharmacological treatments for BD have improved its clinical outlook, many patients do not achieve remission, particularly those suffering from bipolar depression (5). Bipolar depression is often difficult to treat and is linked to worse interpersonal and occupational functional outcomes (6). In addition, available treatments for BD are associated with a myriad of potential adverse effects, such as cardiovascular changes, extrapyramidal symptoms, metabolic abnormalities and weight gain, which often leads to poor adherence (7, 8). This highlights the need for novel therapeutic agents that can be effective for both phases of the illness with a reduced side effect burden.

The objective of this paper is to review the pharmacological properties and clinical efficacy as well as tolerability profile of the novel therapeutic agent cariprazine in the management of patients with BD, taking into account the latest clinical trials data across all phases of the illness. We also included *post hoc* analyses addressing clinically relevant subgroups and symptom domains in BD.

## Overview of Cariprazine

Cariprazine is a piperazine derivative and works primarily as a partial agonist at the dopamine D2 and D3 receptors (9). Compared to aripiprazole and brexpiprazole, which are also partial agonists at the D2 and D3 receptors, cariprazine displays greater selectivity for D3 receptors (10). Cariprazine has been approved by the US Food and Drug Administration (FDA) for the treatment of schizophrenia as well as for the management of manic, mixed and depressive episodes associated with bipolar I disorder in adults. The only other medication with FDA-approval for both mania and bipolar depression is quetiapine. The 2018 Canadian Network for Mood and Anxiety Treatments (CANMAT) and International Society for Bipolar Disorders (ISBD) guidelines recommend cariprazine as a first-line treatment for acute mania, and a second-line treatment for bipolar I depression (11). However, there was insufficient evidence to recommend its use as a maintenance treatment in bipolar I disorder. A recent meta-analysis found that cariprazine was efficacious and safe for the treatment of acute manic, mixed and depressive episodes associated with BD, but that the effect sizes were smaller for bipolar depression (12).

### Pharmacodynamics

Cariprazine is a partial agonist at D2 and D3 receptors, with a 10-fold higher affinity for D3 compared to D2 receptors *in vitro* (13). The activity of cariprazine *in vivo* depends on the functional status of the dopaminergic system; when dopamine activity is normal, it acts as an antagonist and when dopamine tone is low, it becomes a partial agonist (9). Since it has a higher potency for D3 receptors than dopamine, cariprazine administration results in a net effect of D3 antagonism (14). This property differentiates cariprazine from other atypical antipsychotics and may explain its unique pharmacological profile. D3 receptors are primarily located in the ventral tegmental area (VTA), substantia nigra, hypothalamus and limbic areas (15). Due to its lower potential for inhibiting dopaminergic neurotransmission in the striatum, cariprazine may cause less extrapyramidal symptoms (EPS) than other antipsychotics (13). D3 receptors are believed to be involved in locomotor control, cognition and drug abuse (16, 17). As a result, cariprazine has been associated with pro-cognitive effects and improvement in negative symptoms in patients with schizophrenia (18, 19). Cariprazine also demonstrated anti-abuse potential by reducing the rewarding effect of cocaine in rats (20). Since BD is frequently linked to substance use disorders (21), this finding warrants further investigation in clinical trials. Animal models have suggested a role of D3 receptors in mood regulation, and that partial agonism at D3 receptors may have antidepressant effects (22). A possible mechanism of action is that partial agonism/antagonism at D3 receptors inhibit the activity of somatodendritic D3 receptors in the VTA, resulting in increased dopamine release in the prefrontal cortex and subsequent mood improvement (23).

In addition to being a D2 and D3 partial agonist, cariprazine is a strong antagonist at the serotonin 5-HT_2B_ receptors (24), although the clinical relevance of this effect remains unclear. It also has high affinity for the α-_1B_ receptor, which has been associated with reduced EPS and akathisia (25). Cariprazine is a partial agonist at the 5-HT_1A_ receptors and binds to them with moderate affinity; this property may contribute to its antidepressant effects (26). Furthermore, at higher doses of cariprazine, 5-HT_1A_ partial agonism is hypothetically linked to reduced EPS (13). Cariprazine has low to moderate affinity for the 5-HT_2A_, 5-HT_2C_ and H_1_ receptors (9). Due to its relatively weak antagonism at the 5-HT_2C_ and H_1_ receptors, cariprazine may be associated with less sedation, weight gain and metabolic abnormalities compared to other atypical antipsychotics, such as olanzapine and quetiapine. It has negligible activity on cholinergic muscarinic receptors and thus, does not cause anticholinergic side effects (13).

### Pharmacokinetics

Cariprazine is an oral medication that is dosed once-daily, with or without food (27). Following oral administration, cariprazine is rapidly absorbed and reaches peak concentrations within 3–5 h (28). It has a large volume of distribution and is extensively distributed in tissues (29). Cariprazine is primarily metabolized by the CYP3A4 isoenzyme and to a lesser extent, by CYP2D6 into two major active metabolites, desmethylcariprazine (DCAR) and didesmethylcariprazine (DDCAR) (30). Both metabolites are pharmacologically equipotent to cariprazine and appear to mediate its therapeutic effect (31). The mean half-lives of cariprazine and its metabolites are 2–4 days and 1–3 weeks, respectively (32). The long half-life of cariprazine and its metabolites has important clinical implications. After initiating cariprazine, the effective dose may be increasing for many weeks even if the daily dose remains constant, since it takes longer for cariprazine to achieve a steady-state (14). For the same reason, occasionally missing a dose of cariprazine will probably not lead to symptomatic relapse or discontinuation symptoms, as opposed to compounds with shorter half-lives (14). According to a 12-week study, steady-state plasma concentrations were reached within 1–2 weeks for cariprazine and DCAR, and within 4 weeks for DDCAR due to its slow elimination (33). Cariprazine and its metabolites are minimally excreted in urine (29). Cariprazine itself is a weak inhibitor of CYP3A4 and CYP2D6 with no significant induction effects in human hepatocytes (30). The pharmacokinetics properties of cariprazine and its metabolites are not altered by age, sex, race, smoking status, and in patients with mild to moderate hepatic or renal impairment.

### Dosing

For acute mania, the recommended dose range of cariprazine is 3–6 mg/day (34). The recommendation is to start at 1.5 mg/day on day 1 and increase to 3 mg/day as early as on day 2. The dose can be further increased by 1.5–3 mg increments depending on clinical response and tolerability, up to a maximum of 6 mg/day. For bipolar depression, the recommended dose range is 1.5–3 mg/day. The starting dose is 1.5 mg/day, which is the therapeutic dose for the majority of patients. The dose can be increased to 3 mg/day in those that have had a partial response with response plateaued for 2 weeks or longer depending on the tolerability.

Although cariprazine has been studied as a monotherapy agent in bipolar disorder, it can be administered concurrently with lithium, valproic acid or lamotrigine with no dose adjustment required. If cariprazine is used in conjunction with carbamazepine, clinicians need to be aware of the potential reduction in cariprazine levels given that carbamazepine is a potent CYP3A4 inducer; hence, the dose of cariprazine may need to be adjusted accordingly to achieve the desired clinical outcomes. The dose of cariprazine should be reduced by 50% when administered concurrently with strong CYP3A4 inhibitors (27).

## Overview of Clinical Trials and *post hoc* Analyses

The authors searched for clinical trials and *post hoc* analyses published in the PubMed, Web of Science, Embase, PsychInfo and ClinicalTrials.gov electronic databases. The search strategy contained the following terms: cariprazine, bipolar disorder, bipolar affective disorder, mania, bipolar depression and mixed episode. Randomized, double-blind, placebo controlled (RCTs) trials and *post hoc* analyses were included. To look for additional studies that may have not been captured by the original database search, we performed backwards reference chaining by searching through bibliographies of relevant articles.

At the time of this writing, there were three ongoing clinical trials on the efficacy and/or safety of cariprazine in BD registered on ClinicalTrials.gov; one RCT on the efficacy and safety of cariprazine in bipolar I depression in pediatric participants (NCT04777357), one open-label study on the long-term (26-weeks) safety and tolerability of cariprazine in pediatric participants with schizophrenia or bipolar I disorder (NCT04578756), and one RCT on relapse prevention in bipolar I patients with manic or depressive episodes, with or without mixed features (NCT03573297). However, the preliminary results of these trials are currently not available.

The efficacy and safety of cariprazine in treating bipolar I disorder in adults have been examined in seven RCTs. Their results are summarized below.

## Clinical Efficacy

### Efficacy in Mania

Three RCTs (one phase II and two phase III studies) have assessed the efficacy of cariprazine in patients with mania (35–37) (see Table 1).

**Table 1:** Summary of Mania RCTs.

**Reference**	**Intervention**	**Dosing**	**Sample Size^a^**	**YMRS LSMD (95% CI)**	**Response^b^ (%)**	**Remission^c^ (%)**
Durgam et al. (35)	Cariprazine 3–12 mg/day	Flexible	118	−6.1 (−8.9 to −3.3)	48	42
				*p < * 0.0001	*p < * 0.001	*p* = 0.002
	Placebo		117	–	25	23
Calabrese et al. (36)	Cariprazine 3–6 mg/day	Fixed/flexible	165	−6.1 (−8.4 to −3.8)	60.6	44.8
				*p < * 0.001	*p < * 0.001	*p* = 0.003
	Cariprazine 6–12 mg/day		167	−5.9 (−8.2 to −3.6)	59.3	44.3
				*p < * 0.001	*p < * 0.001	*p* = 0.005
	Placebo		160	–	37.5	29.4
Sachs et al. (37)	Cariprazine 3–12 mg/day	Flexible	158	−4.3 (−6.7 to −1.9)	58.9	51.9
				*p* = 0.0004	*p* = 0.0097	*p* = 0.0025
	Placebo		152	–	44.1	34.9

a *Intention-to-treat population*.

b *≥50% YMRS score reduction from baseline*.

c *YMRS total score ≤ 12*.

*YMRS, Young Mania Rating Scale; LSMD, least square mean difference; CI, confidence interval*.

The Durgam et al. trial was a phase 2, multinational, flexible-dose RCT comprising of 235 patients in the intention-to-treat (ITT) population (35). Participants were randomized to receive cariprazine 3–12 mg/day, or placebo for 3 weeks. The mean daily dose of cariprazine was 8.8 mg/day. For the primary parameter, the change in Young Mania Rating Scale (YMRS) total score from baseline to week 3 was significantly greater for the cariprazine group compared to the placebo group [least square mean differences [LSMD]: −7.0 (95% confidence interval [CI]: −10.0 to −4.0; *p* < 0.0001)] using a mixed-effects model for repeated measures (MMRM) approach. The improvement in YMRS score was observed on day 7, and was maintained through the end of the trial. Cariprazine-treated patients had significantly higher response and remission rates compared to placebo-treated patients, with number needed to treat (NNT) estimates of 5 for response and 6 for remission.

Calabrese et al. conducted a phase 3, multicenter, fixed/flexible-dose RCT, which included 492 patients in the ITT population (36). Participants were randomly assigned to cariprazine 3–6 mg/day (low dose), cariprazine 6–12 mg/day (high dose), or placebo for 3 weeks. The mean daily doses were 4.8 mg for the 3–6 mg/day group, and 9.1 mg for the 6–12 mg/day group. For the primary efficacy measure, the YMRS total change score from baseline to week 3 was significantly greater for both cariprazine groups compared to placebo [LSMD for the 3–6 mg/day group: −6.1 (95% CI: −8.4 to −3.8; *p* < 0.001); LSMD for the 6–12 mg/day group: −5.9 (95% CI: −8.2 to −3.6; *p* < 0.001)]. *Post hoc* analyses of the primary outcome resulted in effect sizes of 0.62 for the 3–6 mg/day group, and 0.60 for the 6–12 mg/day group using a MMRM approach. In addition, both cariprazine groups were associated with significantly higher response and remission rates compared to placebo, with NNT estimates of 5 for response and 7 for remission in both groups.

The Sachs et al. study was a phase 3, flexible-dose RCT involving 310 participants in the ITT population (37). Similar to the Durgam et al. trial, patients were randomized to receive cariprazine 3–12 mg/day, or placebo for 3 weeks. For the primary outcome, the cariprazine group had a significantly greater reduction in YMRS total score from baseline to week 3 compared to placebo [LSMD: −4.3 (95% CI: −6.7 to −1.9; *p* = 0.0004)]. The improvement in YMRS score was observed as early as 4 days after the baseline assessment, and was maintained until the end of the trial. The effect size for cariprazine on YMRS change score was 0.45 using a MMRM approach. Response (58.9 vs. 44.1%) and remission (51.9 vs. 34.9%) rates were significantly higher in the cariprazine-treated patients vs. placebo-treated patients.

### Efficacy in Bipolar Depression

Four RCTs (two phase II and two phase III studies) have evaluated the efficacy of cariprazine in participants with bipolar depression (38–41) (see Table 2).

**Table 2:** Summary of Bipolar Depression RCTs.

**Reference**	**Intervention**	**Dosing**	**Sample size^a^**	**MADRS LSMD (95% CI)**	**HAMD-17 LSMD (95% CI)**	**Response^b^ (%)**	**Remission^c^ (%)**
Yatham et al. (38)	Cariprazine 0.25–0.75 mg/day	Fixed/flexible	64	−0.7 (−4.6 to 3.3)	−0.2 (−2.9 to 2.5)	56.0	48.0
				*p* = 0.7408	*p* = 0.8936	*p* = 0.4026	*p* = 0.2298
	Cariprazine 1.5-3 mg/day		54	0.0 (−4.1 to 4.1)	0.2 (−2.6 to 3.0)	54.1	43.2
				*p* = 0.9961	*p* = 0.8891	*p* = 0.5066	*p* = 0.4512
	Placebo		60	–	–	49.3	38.7
Durgam et al. (39)	Cariprazine 0.75 mg/day	Fixed	140	−1.9 (−4.3 to −0.5)	−1.1 (−2.9 to 0.6)	38.6	23.6
				*p* = 0.129	*p* = 0.199	*p* = 0.227	*p* = 0.340
	Cariprazine 1.5 mg/day		145	−4.0 (−6.3 to −1.6)	−2.7 (−4.4 to −1.0)	49.7	36.6
				*p* = 0.003	*p* = 0.002	*p* = 0.002	*p* = 0.002
	Cariprazine 3 mg/day		145	−2.5 (−4.9 to −0.1)	−2.2 (−3.9 to −0.5)	44.8	27.6
				*p* = 0.112	*p* = 0.013	*p* = 0.024	*p* = 0.105
	Placebo		141	–	–	31.9	19.9
Earley et al. (40)	Cariprazine 1.5 mg/day	Fixed	162	−2.5 (−4.6 to −0.4)	−1.6 (−3.2 to 0.1)	40.7	25.9
				*p* = 0.0417	*p* = 0.0590	*p* = 0.3383	*p* = 0.1648
	Cariprazine 3 mg/day		153	−1.8 (−3.9 to 0.4)	−0.5 (−2.1 to 1.2)	42.5	26.1
				*p* = 0.1051	*p* = 0.5599	*p* = 0.2088	*p* = 0.1625
	Placebo		163	–	–	35.6	19.6
Earley et al. (41)	Cariprazine 1.5 mg/day	Fixed	154	−2.5 (−4.6 to −0.4)	−2.4 (−4.0 to −0.8)	48.1	33.1
				*p* = 0.0331	*p* = 0.0042	*p* = 0.1300	*p* = 0.0374
	Cariprazine 3 mg/day		164	−3.0 (−5.1 to −0.9)	−1.3 (−3.0 to 0.3)	51.8	32.3
				*p* = 0.0103	*p* = 0.0996	*p* = 0.0243	*p* = 0.0391
	Placebo		156	–	–	39.7	23.1

a *Intention-to-treat population*.

b *≥50% MADRS score reduction from baseline*.

c *MADRS total score ≤ 10*.

*MADRS, Montgomery-Åsberg Depression Rating Scale; HAMD-17, 17-item Hamilton Depression Rating Scale; LSMD, least square mean difference; CI, confidence interval*.

Yatham et al. led a phase 2, fixed/flexible-dose RCT, which included 224 participants in the ITT population (38). This trial recruited patients with both bipolar I and bipolar II disorder, who were randomly allocated to cariprazine 0.25–0.5 mg/day (low dose), cariprazine 1.5–3.0 mg/day (high dose), or placebo for 8 weeks. Neither cariprazine group significantly separated from placebo for the primary (change in Montgomery-Åsberg Depression Rating Scale [MADRS] total score from baseline to week 8) and secondary (change in Clinical Global Impressions—Severity/Improvement [CGI-S, CGI-I], 17-item Hamilton Depression Rating Scale [HAMD-17] and 24-item HAMD [HAMD-24] scores) efficacy measures. In addition, there was no significant difference in response and remission rates between both cariprazine groups and placebo.

Durgam and colleagues conducted a phase 2, multicenter, fixed-dose trial which included 571 patients in the ITT population (39). Participants were randomized to receive cariprazine 0.75 mg/day, cariprazine 1.5 mg/day, cariprazine 3.0 mg/day, or placebo. For the primary efficacy outcome, the MADRS score change from baseline to week 6 was statistically significant only for the cariprazine 1.5 mg/day group [LSMD: −4.0 (95% CI: −6.3 to −1.6; adjusted *p* = 0.003)]. The cariprazine 0.75 and 3.0 mg/day groups failed to separate from placebo, although the 3.0 mg/day group demonstrated a greater MADRS score reduction than placebo (adjusted *p* = 0.112). The MADRS effect sizes were 0.20, 0.42, and 0.26 for the cariprazine 0.75, 1.5, and 3.0 mg/day groups, respectively. Only the cariprazine 1.5 mg/day group had significantly higher rates of MADRS response (NNT = 6, *p* < 0.05) and remission (NNT = 6, *p* < 0.01) compared to placebo. The cariprazine 3.0 mg/day group was significantly superior to placebo only for the MADRS response rate (NNT = 8, *p* < 0.05).

Earley et al. conducted a phase 3, fixed-dose RCT involving 478 participants in the ITT population (40). Patients were randomized to cariprazine 1.5 mg/day, cariprazine 3.0 mg/day, or placebo for 6 weeks. For the primary efficacy outcome, only the cariprazine 1.5 mg/day group demonstrated a statistically significant reduction in MADRS total score from baseline to week 6 compared to placebo [LSMD: −2.5 (95% CI: −4.6 to −0.4; adjusted *p* = 0.0417)]. Although patients who received cariprazine 3.0 mg/day had a lower MADRS total score at week 6 vs. placebo, the difference did not reach statistical significance (*p* = 0.1051). The effect sizes for cariprazine at week 6 were 0.28 for the cariprazine 1.5 mg/day group, and 0.20 for the 3.0 mg/day group. Both cariprazine groups failed to achieve higher response and remission rates compared to placebo using the MADRS criteria. However, a significantly greater percentage of patients in the cariprazine 1.5 mg/day group met criteria for HAMD-17 remission (30.6 vs. 16.4%, *p* = 0.0051), which was not the case for the cariprazine 3.0 mg/day group.

Finally, in a phase 3, fixed-dose RCT comprising of 475 patients in the ITT population, participants were randomly assigned to receive cariprazine 1.5 mg/day, cariprazine 3.0 mg/day, or placebo (41). For the primary efficacy parameter, both cariprazine groups showed a significant reduction in MADRS total scores from baseline to week 6 [LSMD for the 1.5 mg/day group: −2.5 (95% CI: −4.6 to −0.4; adjusted *p* = 0.033); LSMD for the 3.0 mg/day group: −3.0 (95% CI: −5.1 to −0.9; *p* = 0.010)]. The effect sizes were 0.28 and 0.34 for the cariprazine 1.5 and 3.0 mg/day groups, respectively. MADRS response rates only reached statistical significance for the cariprazine 3.0 mg/day group (51.8%, NNT = 8, *p* = 0.024). However, MADRS remission rates were significantly higher in both cariprazine groups (33.1%, NNT = 10, *p* = 0.037 for the 1.5 mg/day group; and 32.3%, NNT=11, *p* = 0.039 for the 3.0 mg/day group).

## Safety and Tolerability

Cariprazine was shown to be generally well-tolerated in the RCTs for BD. The majority of treatment-emergent adverse events (TEAEs; defined as ≥5% in the cariprazine group(s) and twice the rate of placebo) were mild or moderate in intensity.

Two *post hoc* analyses have assessed the safety and tolerability of cariprazine by pooling the data from the above RCTs; one *post hoc* analysis was carried out in patients with acute manic/mixed episodes (42), while the other was performed in participants with bipolar depression (43). For the *post hoc* analysis of mania, cariprazine was associated with more TEAEs compared to placebo, but the majority of adverse events were mild to moderate in severity (42). The most common cariprazine TEAEs were akathisia, extrapyramidal symptoms, restlessness and vomiting. The incidence of adverse events leading to study discontinuation was higher in cariprazine compared to placebo, with akathisia being the most common reason. Overall, there were no significant differences for clinically meaningful weight gain (≥7% increase in body weight) or mean change from baseline in metabolic parameters between cariprazine and placebo. Clinically significant weight gain occurred at a similar rate between cariprazine (1.9%) and placebo (1.6%). The mean increases in fasting glucose levels were higher in cariprazine (7.0 mg/dL) than placebo-treated patients (1.7 mg/dL). Cariprazine was not associated with any clinically meaningful changes in electrocardiogram parameters or prolactin levels.

For the *post hoc* analysis of bipolar depression, TEAEs occurred at a similar rate between cariprazine (60%) and placebo-treated (55%) patients (43). The most common TEAEs associated with cariprazine use were akathisia and nausea. Treatment-emergent akathisia occurred in 9.9% of cariprazine and 4.3% in placebo-treated patients. Adverse events leading to discontinuation were slightly higher in the cariprazine group, and the most common side effect leading to discontinuation was akathisia. Similar to the *post hoc* analysis of mania, cariprazine did not lead to clinically significant weight gain or changes in metabolic parameters compared to placebo. The mean weight gain was <1 kg for cariprazine-treated patients. The mean increase in fasting glucose levels was similar for cariprazine (3.1 mg/dL) and placebo (2.6 mg/dL). Cariprazine did not led to any significant changes in electrocardiogram parameters or prolactin levels relative to placebo.

A separate *post hoc* analysis of treatment-emergent akathisia in patients with bipolar I depression showed that the overall incidence of akathisia in cariprazine-treated patients was 7.6% (compared to 2.1% for placebo), which was dose-dependent (44). Akathisia occurred during the first 3 weeks, and was generally mild to moderate in severity, rarely leading to treatment discontinuation. Akathisia associated with cariprazine use can be managed by adding a beta-blocking agent, such as propranolol. In the same *post hoc* analysis, 23.5% of cariprazine-treated patients who achieved akathisia resolution during treatment received a beta-blocking medication (44)”.

## *Post hoc* Analyses

### Broad Spectrum Efficacy of Cariprazine

The broad spectrum efficacy of cariprazine has been assessed in two *post hoc* analyses. Vieta et al. pooled data from three positive RCTs (35–37) to assess the efficacy of cariprazine 3–12 mg/day on manic symptoms in adult patients with bipolar I disorder (45). Cariprazine was associated with a significant improvement on all 11 individual YMRS symptom items (*p* < 0.001), with the largest effect sizes for irritability (0.55) and disruptive-aggressive behavior (0.49). In addition, significantly more cariprazine-treated patients had mild or no symptoms on all YMRS items at the end of the double-blind phase (*p* < 0.001).

In another *post hoc* analysis, data from three bipolar depression RCTs (39–41) were pooled to explore the efficacy of cariprazine in bipolar depression (46). The data was analyzed in combined cariprazine (pooled 1.5 and 3 mg/day) and individual dose groups (1.5 or 3 mg/day). The cariprazine 1.5–3, 1.5, and 3 mg/day groups were all associated with a significant reduction in MADRS total score from baseline to week 6 (LSMD = −2.6 for 1.5–3 mg/day; −2.8 for 1.5 mg/day; −2.4 for 3 mg/day; *p* < 0.001 for all dose groups). The combined cariprazine 1.5–3 mg/day group showed a significant improvement on all individual MADRS items, except for inner tension. In addition, the combined and 3 mg/day groups had significantly lower scores on the suicidal thoughts item at the end of treatment, but the mean changes were overall small.

### Mania With Mixed Features

In this *post hoc* analysis pooling data from the three mania RCTs (35–37), McIntyre et al. used three criteria to define mania with mixed features: ≥3 depressive symptoms (DS) using the DSM-5, ≥2 DS and MADRS total score ≥10 (47). Overall, cariprazine was associated with significant improvement in mean YMRS scores compared to placebo for each criterion (LSMD = −3.79, *p* = 0.0248 for ≥3 DS; −2.91, *p* = 0.0207 for ≥2 DS; −5.49, *p* < 0.0001 for MADRS ≥10). In addition, more cariprazine- than placebo-treated patients met YMRS response and remission criteria, reaching statistical significance in the ≥2 DS and MADRS ≥10 subgroups.

### Bipolar Depression With Mixed Features

This *post hoc* analysis by McIntyre et al., which pooled data from three bipolar depression RCTs (39–41), used a baseline YMRS total score ≥4 to characterize depressed patients with concurrent manic symptoms (48). In that subgroup, both cariprazine 1.5 and 3.0 mg/day groups had significant reductions in MADRS total score from baseline to week 6 relative to placebo (LSMD = −2.5, *p* = 0.0033 for 1.5 mg/day; −2.9, *p* = 0.0010 for 3.0 mg/day).

### Cognition in Mania

McIntyre et al. pooled data from the three mania RCTs (35–37) in this *post hoc* analysis to assess its effects on cognition (49). Cognitive symptoms were evaluated using the Positive and Negative Syndrome Scale (PANSS) Cognitive subscale, with a baseline score ≥15 indicating cognitive symptoms. In patients with baseline cognitive symptoms, cariprazine-treated patients showed significantly greater mean improvement on PANSS cognitive subscale scores compared to placebo (−4.0 vs. −1.9, *p* = 0.0002).

### Functioning in Bipolar Depression

Vieta et al. carried out a *post hoc* analysis of a cariprazine RCT (39) to assess its efficacy on function in bipolar I depression (50). The authors used the mean changes from baseline to week 8 in Functional Assessment Short Test (FAST) total score as a measure of functioning. The FAST is a 24-item scale which assesses functioning in the following areas: autonomy, occupational functioning, cognitive functioning, financial issues, interpersonal relationships and leisure time. The FAST total score ranges from 0 to 72, with a higher score indicating worse functioning. The cariprazine 1.5 mg/day group had a statistically significant reduction in FAST total score from baseline to week 8 compared to placebo (LSMD = −5.3, *p* = 0.0051), but not the 3.0 mg/day group (LSMD = −3.2, *p* = 0.0575). In addition, the LSMD favored cariprazine 1.5 mg/day on all the FAST subscales except financial issues (*p* < 0.05). Rates of functional remission (FAST total score ≤ 20) and recovery (FAST total score ≤ 11) at week 8 were significantly greater for cariprazine 1.5 mg/day relative to placebo.

## Discussion

Cariprazine is a partial agonist at the D2 and D3 receptors with a greater selectivity for D3 receptors, which makes it unique among atypical antipsychotics. At doses ranging from 3 to 12 mg/day, cariprazine was effective in the treatment of manic episodes. It was associated with a significant reduction in YMRS total scores with a moderate effect size. In addition, cariprazine-treated patients achieved significantly higher response and remission rates compared to placebo, with NNT estimates of 5–7. However, doses above 6 mg/day did not appear to confer any additional benefit, except in the Calabrese et al. study, where the cariprazine 6–12 mg/day group did better when the cutoff for remission was set at YMRS ≤ 8 (a stricter cutoff than the usual definition of YMRS ≤ 12) (36). Cariprazine was also effective in mania with mixed features according to a recent *post hoc* analysis (47).

For bipolar depression with or without mixed features, cariprazine also demonstrated clinical efficacy at doses of 1.5–3.0 mg/day, while doses below 1.5 mg/day were clearly ineffective as shown by the Durgam et al. trial (39). Although both cariprazine 1.5 and 3.0 mg/day doses were associated with significant reduction in MADRS total scores, the effect sizes were small and lower than in the trials for mania. Response and remission rates were overall higher in cariprazine-treated patients compared to placebo, with NNT estimates of 6–10. Interestingly, the 3 mg/day dose did not provide much additional clinical efficacy compared to 1.5 mg/day, and was associated with higher discontinuation rates.

Cariprazine was overall well-tolerated, and the majority of TEAEs were mild or moderate in intensity. The incidence of TEAEs appeared to be dose-dependent. In both mania and depression trials, cariprazine was associated with more adverse events than placebo, with the most common side effects being akathisia, extrapyramidal symptoms and nausea. Akathisia was the main adverse event leading to discontinuation in cariprazine-treated patients. However, cariprazine was not associated with clinically meaningful changes in body weight, metabolic parameters or prolactin levels.

Although there are no head-to-head trials comparing cariprazine to other atypical antipsychotics, cariprazine appears to be equally effective as other antipsychotics for the treatment of mania, with a better tolerability profile. The NNT estimates of 5–7 for cariprazine response are in keeping with the results of a comparative analysis which assessed the efficacy of antipsychotics commonly used in the treatment of mania, such as aripiprazole, olanzapine, quetiapine, risperidone and ziprasidone (pooled NNTs of 6 for reduction in YRMS scores) (51). For bipolar I depression, the only atypical antipsychotics recommended as first-line treatments by the CANMAT and ISBD guidelines are quetiapine and lurasidone (11). The NNT estimate of 10 for cariprazine remission is higher than the NNTs for quetiapine (NNT of 6) and lurasidone (NNT of 5), but compares favorably to adjunctive antidepressants (NNT of 14) (12). However, the criteria used for defining remission in cariprazine studies was more stringent (i.e., MADRS score ≤ 10) compared with quetiapine and lurasidone studies which used a cut off score of ≤ 12; thus, direct comparisons of remission rates are not appropriate. Since up to 54% of patients with BD meet criteria for metabolic syndrome (52), the need for metabolic-neutral agents for the management of BD is of paramount importance. Lurasidone, despite its efficacy in bipolar I depression, has not been studied in mania and is associated with extrapyramidal symptoms like akathisia (53). In contrast, aripiprazole monotherapy is effective for the treatment of manic episodes, but not for bipolar depression (54). Considering its broad spectrum efficacy for both phases of the illness and favorable tolerability profile, cariprazine has the potential to provide an important new option among atypical antipsychotics in the treatment of BD.

Preclinical studies have suggested that cariprazine may enhance cognition in mice by improving attention and memory (55). Although no study to date has assessed the efficacy of cariprazine in improving cognitive function in bipolar patients, there is some evidence from a *post hoc* analysis that cariprazine is associated with an improvement in cognitive symptoms in patients with mania (49). In addition, there is preliminary evidence that lurasidone adjunctive therapy is more effective than treatment as usual in improving global cognition in euthymic patients with bipolar I disorder (56). This has important clinical implications since patients with BD display broad cognitive impairments that persist even during periods of euthymia (57). Our group is currently recruiting participants for a proof of concept, double blind RCT to assess the efficacy of cariprazine in improving cognition in euthymic patients with bipolar I disorder (NCT04771299).

## Conclusion

In conclusion, cariprazine monotherapy has shown efficacy as well as a good tolerability and safety profile for the acute treatment for manic, mixed and depressive episodes associated with BD. *Post hoc* analyses also support its efficacy for mania and bipolar depression with mixed features. Cariprazine appears to have procognitive effects in preclinical studies, but this needs to be examined in larger clinical trials. Future research directions should include studies on relapse prevention, as well as head-to-head trials comparing cariprazine to other atypical antipsychotics with established efficacy in bipolar disorder.

## Author Contributions

AD conducted a review of the literature and drafted the manuscript. KK, AS, and LY reviewed the manuscript. All authors contributed to the article and approved the submitted version.

## Conflict of Interest

AD was partly supported by an unrestricted fellowship grant from Janssen Canada. KK has no disclosures. AS has been on speaker/advisory boards for, or has received research grants from Abbvie, Allergan, CANMAT, CIHR, Janssen, and Otsuka. LY has been on speaker/advisory boards for, or has received research grants from Abbvie, Alkermes, Allergan, CANMAT, CIHR, Dainippon Sumitomo Pharma, Intracellular Therapies, Lundbeck, Merck, Otsuka, Sanofi, and Sunovion.

## Publisher's Note

All claims expressed in this article are solely those of the authors and do not necessarily represent those of their affiliated organizations, or those of the publisher, the editors and the reviewers. Any product that may be evaluated in this article, or claim that may be made by its manufacturer, is not guaranteed or endorsed by the publisher.
